# The Performance of the Iranian Red Crescent by Launching Testing Centers for the Coronavirus Disease

**DOI:** 10.1017/dmp.2020.167

**Published:** 2020-05-22

**Authors:** Hamed Seddighi

**Affiliations:** Student Research Committee, University of Social Welfare and Rehabilitation Sciences, Tehran, Iran; Education and Research, Iranian Red Crescent Society, Yazd Province, Yazd, Iran

**Keywords:** COVID-19, fever detection, screening, volunteering

## Abstract

The coronavirus disease (COVID-19) screening project has been conducted by the Red Crescent Society for 17 days with the aim of identifying and treating people with COVID-19, reducing road trips, and sensitizing people to the problem. Due to the shortage of testing devices, passengers were screened by measuring skin temperature. In 851 screening posts, 95 371 volunteers of Red Crescent monitored 21 640 866 people.

Besides screening people, the stations have had other functions, including urging people to stay at home, restricting road trips, especially during the Iranian New Year holidays, reassuring the people that all relevant organizations are doing their best to respond to COVID-19, and the like. However, future research is still needed to analyze the cost-benefit of this plan and other possible options.

Iran announced the first confirmed cases of severe acute respiratory syndrome coronavirus 2 (SARS-CoV-2) infection on February 19, 2020, during the 2019–2020 coronavirus pandemic.^1,2^ Iran has a high number of coronavirus disease (COVID-19) cases in the world and has been the worst affected country in the Middle East until May 2, 2020.^3^ The total number of people living with COVID-19 in Iran so far is 96 448, death toll reached 6156, and the total number of people who recovered from the disease so far is 77 350.^4^

The Iranian Red Crescent Society (IRCS) is a member of the Coronavirus Response Headquarters in Iran. During division of work among members of the headquarters, 1 of the tasks assigned to IRCS was establishing testing centers using the large number of volunteers. Coronavirus Testing Centers or mobile fever detection stations were launched on March 18, 2020, in Iran.

## NARRATIVE

The screening program began on March 18 in several provinces and expanded within a week. In this plan, the IRCS operational forces, based on the situation in provinces, worked in more than 851 temporary stations or posts at the entrances and exits of cities, train stations, and airports to control passengers and identify the suspected cases of COVID-19. In each post, 4 trained personnel consisting of aid workers (volunteer and staff) of IRCS screened passengers for COVID-19 symptoms and referred the people with the symptoms to the specified health centers until the end of April 4, 2020.^5^

Other stakeholders of IRCS in this plan included the Ministry of Health, the Judiciary, Police, and Ministry of Roads and Urban Development under the command of the governor of the province or county. During the implementation of the plan, 21 640 861 people were screened. The number of people screened and reported by the IRCS has been estimated based on the number of passenger cars and the assumption of 3 passengers per car. Screening was performed only by using thermometers.^5^ The Red Crescent Society stated that approximately 14 302 car passengers screened had symptoms of high fever and were suspected to have COVID-19.

Every day, 4930 volunteers and employees in the IRCS screening stations in different provinces of the country screened the people. The plan was implemented completely through 95 371 person-days and 15 278 vehicle-days. Due to the high number of patients in 13 provinces, including Tehran, Qazvin, Razavi Khorasan, Gilan, Golestan, Mazandaran, Qom, Markazi, Isfahan, Semnan, Hamedan, Alborz, and Lorestan, the number of posts in those regions is higher than other provinces. The stations provided service for 24 hours a day.^5^

At the stations, the screening job was assigned to IRCS volunteers and, once symptoms were identified, the suspected individual was referred to treatment experts. IRCS was not responsible for transferring patients to Corona Regional Medical Center. Also, in order to prevent any resistance or conflict by suspected or known patients, the military and law enforcement forces were present at the place along with the IRCS aid workers and the regional health network.

All candidates attending the plan had previously completed first aid training courses and prehospital emergency training for 22 hours and 45 hours, respectively. To train volunteers for screening, first a 4-hour online course on coronavirus was presented, and then the necessary information was provided through a brochure. Also, older staff in posts gave instructions to the newcomers. Attending the posts was voluntary and unpaid, but the cost of food (breakfast, lunch, dinner) was paid to the branches by Red Crescent. The minimum age required for taking such positions was 18 years with no history of serious disease. The shifts were rotating every 6 hours, and new people were substituted for the previous shift in each post.

## DISCUSSION

Twenty volunteers reportedly have been infected with SARS-CoV-2 and then recovered from the disease. At the beginning of the project, IRCS provided health insurance coverage to volunteer aid workers so that any medical expense, in case of infection, would be borne by the insurance company as the party to the contract with Red Crescent. In all cities and counties, the volunteers were examined by a doctor at 5-day intervals in order to ensure their health and safety. Also, if any symptom of COVID-19 was recognized in a volunteer, that volunteer would be urgently referred to the medical doctor.

As IRCS responds to the coronavirus, other natural disasters, especially floods, are affecting various provinces in Iran. There has been public concern that the IRCS would not be able to respond to other disasters while focusing on the COVID-19 screening project. However, the IRCS that the plan did not support using special volunteer forces disaster experts and instead it used other volunteers for screening. IRCS also has roadside, mountain, and sea relief bases across the country to respond to road accidents, as well as mountain and sea hazards.^6,7^ In order to keep enough capacity to respond to such incidents, IRCS ordered the provinces not to use their aid workers present in the above bases for the screening plan so that, in case of an accident, the rescue forces would be able to respond.

## CONCLUSION

The IRCS used a significant number of volunteers and facilities for screening. Due to a lack of COVID-19 testing devices, the screening job was performed by only measuring skin temperature and, sometimes, with pulse oximeters. Measuring body temperature or fever detection is not a reliable way to identify people with COVID-19. Therefore, supposing that the organizers of this project were aware of that fact, then they probably have had other purposes to conduct such a plan, including sensitizing and urging people to stay at home, enforcing road restrictions, especially during the Iranian New Year holidays, and reassuring the people that all relevant organizations are doing the best to respond to COVID-19 rather than actually identifying the patients. However, future research is still needed to investigate the cost-benefit of this activity and alternative options.
